# Changes in Interhemispheric Motor Connectivity Across the Lifespan: A Combined TMS and DTI Study

**DOI:** 10.3389/fnagi.2019.00012

**Published:** 2019-02-05

**Authors:** Sebastian Strauss, Martin Lotze, Agnes Flöel, Martin Domin, Matthias Grothe

**Affiliations:** ^1^Department of Neurology, University Medicine of Greifswald, Greifswald, Germany; ^2^Functional Imaging, Institute for Diagnostic Radiology and Neuroradiology, University Medicine of Greifswald, Greifswald, Germany

**Keywords:** TMS, DTI, ISP, interhemispheric inhibition, age

## Abstract

Age-related decline in interhemispheric connectivity between motor areas has been reported with both transcranial magnetic stimulation (TMS) and diffusion tensor imaging (DTI) measurements. However, not all studies were able to confirm these findings, and previous studies did not apply structural (DTI) and functional (TMS) measurements within each individual appropriately. Here, we investigated age dependency of the ipsilateral silent period (ISP) and integrity of fibers in the corpus callosum as operationalized by fractional anisotrophy (FA), using TMS and DTI, respectively, in 20 participants between 19 and 72 years of age. We found age-dependent increase for ISP, and decrease of FA, both indicating a decrease in interhemispheric inhibition, with a negative association between FA and ISP for the dominant hemisphere (*r* = −0.39, *p* = 0.043). Our findings suggest that aging leads to decline of interhemispheric motor connectivity, as evidenced in both structural and functional parameters, which should be taken into account when interpreting disease- or medication-related changes.

## Introduction

Motor performance depends on the interaction of a number of cortical and subcortical areas, and even unilateral movements include bilateral primary and secondary motor network activation (Carson, 2005; Vercauteren et al., 2008; Fling and Seidler, 2012). The interhemispheric interaction between homologous primary motor areas (M1) mediated by transcallosal fibers (Carson, 2005; Wahl et al., 2007) is particularly important: this pathway conveys inhibitory signals to modulate and optimize manual dexterity for the upper limbs (Carson, 2005; Cincotta and Ziemann, 2008), and its connectivity is subject to physiological and pathological modulations, such as those seen during long term training (Walz et al., 2015) or the increase of mirror movements following unilateral cortical brain damage (Beaulé et al., 2012).

The interaction between the two M1 areas can be assessed functionally with transcranial magnetic stimulation (TMS) and structurally with diffusion tensor imaging (DTI). For TMS, ipsilateral silent period (ISP) has been most widely used. It is a well-known neurophysiological TMS method to quantify transcallosal inhibitory drive, and can be evaluated with regard to onset, duration, and depth (Ferbert et al., 1992; Chen et al., 2003). An ongoing ipsilateral muscle activity is interrupted by stimulating M1 with a single TMS pulse, which is thought to be mediated by transcallosal inhibition between the stimulated and the contralateral pre-activated M1 (Ferbert et al., 1992; Chen et al., 2003). Age-related decline in this interhemispheric interaction have been reported in a number of studies (reviewed by Levin et al., 2014), and although alterations in onset, duration, and depth parameters have consistently been reported over the lifespan, the parameter optimally reproducing age related changes remains unknown (Levin et al., 2014).

DTI is widely used to assess structural connectivity* in vivo*, most often with fractional anisotropy (FA) as an established method for the quantification of water diffusion orientation (Bonilha et al., 2015) after calculating diffusion tensors, and can be used to evaluate inter-hemispheric connectivity between M1 areas (Lindow et al., 2016). Some DTI-studies confirmed white matter tract changes across the lifespan and emphasized that FA decline is the most sensitive parameter, but with conflicting results for the interhemispheric motor tracts (Sullivan et al., 2010; Inano et al., 2011; Zhao et al., 2015). Such conflicting results might in part be due to difficulties selecting the precise position of the upper limb bilateral M1 interhemispheric tract. Here, technical advances in the parcellation of the corpus callosum, as previously performed for the human connectome project (Domin and Lotze, in press), now enable an optimized differentiation of the upper limb M1-M1 motor connectivity.

Only one previous study has combined TMS and MRI to investigate age-associated changes in interhemispheric connectivity (Fling et al., 2012). Comparing a group of young and old participants, the authors found differential association between the depth of the ISP and the mean FA inter-hemispheric connectivity between both M1 areas as a function of age: the young group showed a positive association between ISP and FA, while in the older participants, a negative association was found. There is no study investigating the relationship between structure and function in a cohort covering the adult lifespan.

In the present study, our aim was threefold: first, we wanted to determine which parameter of functional interhemispheric connectivity (onset, duration, depth of ISP) would show the strongest negative correlation to age.

Second, we aimed to determine which parameter of structural interhemispheric connectivity (M1-M1 interhemispheric connectivity compared to the upper limb subdivision of M1-M1 interhemispheric connectivity) would show the strongest negative correlation to age.

In the final step, we associated the structural and the functional parameter to investigate the functional-structural relationship across the adult lifespan.

## Materials and Methods

### Participants

Twenty healthy participants between 19 and 72 years of age (mean 43.5 ± 18.1 years; 10 female) were included in the group analysis. All were right handed except one [handedness score (Oldfield, 1971) mean 80, range: −80 to 100]. All subjects gave written informed consent in accordance with the Declaration of Helsinki. The protocol was approved by the ethics committee of the University of Greifswald. TMS and MRI data acquisition were not more than 1 month apart. Group characteristics are summarized in Table 1.

**Table 1:** Group characteristics.

Age	Mean 43.5 ± 18.1; range 19–72
Gender	10 male, 10 female
Handedness	19 r, 1 l; OHS 80; range −80 to 100
RMT dominant	48.9 ± 5.4
RMT non-dominant	47.15 ± 8.1
AMT dominant	42.3 ± 6.0
AMT non-dominant	42.8 ± 6.1

*All mean ± standard deviation, handedness: r, right; l, left; OHS, Oldfield handedness score; RMT, resting motor threshold; AMT, active motor threshold, both given in % maximal stimulator output (%MSO)*.

### Electrophysiological Measurements

Electromyographic (EMG) recording of the right and left first dorsal interosseous (FDI) muscle was performed with 10 mm Ag/AgCl electrodes. After standard skin preparation, one electrode was placed on the muscle belly and the other on the tendon. EMG signals were amplified (CED 1902; Cambridge Electronic Design, Cambridge, United Kingdom), band-pass filtered (20–1,000 Hz), sampled at 2 kHz (CED 1401), and stored for offline analysis (Signal V.08, Cambridge Electronic Design, Cambridge, United Kingdom).

Single pulse TMS was delivered to right and left primary motor cortex using a Magstim 200 stimulator (Magstim Company, Dyfed, UK). Magnetic stimuli were presented using a figure-eight coil held tangentially to the scalp at an angel of 45° to induce current flow in a posterior to anterior direction. The coil was located at the optimal site for producing maximal motor evoked potentials (MEPs) in the resting FDI contralateral to the simulation side (Petoe et al., 2013).

Participants sat comfortably with their hands resting on armrests during the TMS investigation. First, resting motor threshold (RMT) and active motor threshold (AMT) were determined following the IFCN guidelines. RMT was defined as the minimum intensity that induced a MEP of least 50 μV peak-to-peak amplitude in 5 of 10 trials. AMT was defined as the minimum intensity for eliciting MEPs (at least 100 μV) in 5 out of 10 trials while the FDI muscle was pre-activated by performing a pinch-grip (Rossini et al., 2015).

Participants were encouraged to perform an isometric pinch-grip of maximal voluntary contraction (MVC) ipsilateral to the simulation side for the ISP protocol (Cincotta et al., 2004; Trompetto et al., 2004; Giovannelli et al., 2009). Steady maintenance of the contraction during stimulation sessions was visually monitored with a variometer and pauses were added if necessary to avoid muscle fatigue. Twenty ipsilateral TMS stimuli were applied while performing active contraction with an intensity of 90% stimulator output to produce MEPs above threshold in the resting contralateral FDI (Coppi et al., 2014). The ISP protocol was performed on both the dominant and non-dominant hemisphere followed by off-line visual data analysis.

Mean pre-trigger EMG of the ipsilateral hand was measured 30–60 ms before stimulus onset after rectifying and averaging the 20 individual trials. ISP measurements consisted of ISP onset (ISP_onset), ISP duration (ISP_duration), ISP depth (ISP_depth), and transcallosal conduction time (TCT). ISP onset was determined at the timepoint when the post-stimulation EMG fell below the pre-trigger EMG at least for 10 ms, and the corresponding offset was defined at the timepoint when activity resumed for at least 10 ms. ISP duration was calculated as the difference between ISP offset and ISP onset (Boroojerdi et al., 1998; Höppner et al., 1999). ISP depth was calculated as the percentage of mean pre-trigger and post-trigger EMG. Depth % = 100 − [(EMG post/EMGpre) × 100].

TCT was calculated by subtracting the mean MEP latency when stimulating the contralateral motor cortex from the ISP onset (Petitjean and Ko, 2013).

### DTI Measurements

A 3T scanner (Verio, Siemens, Erlangen, Germany) with a 32-channel head coil was used for MR imaging. Two sequences were measured with the following parameters: a T1-weighted 3D MPRAGE (voxel size 1 mm^3^; 176 slices; matrix size 256 × 256; TR 1,690 ms; TE 2.52 ms; GRAPPA PAT acceleration factor 2; acquisition time 3:50 min) and a diffusion-weighted EPI (voxel size 1.95 × 1.95 × 2 mm^3^; 70 slices; matrix size 128 × 128; one b0 volume; 64 diffusion directions; *b*-value 1,000 s/mm^2^; TR 11,400 ms; TE 97 ms; flip angle 90°; acquisition time 12 min).

After conversion of the raw diffusion data to the NIFTI format, the FSL (v5.0.6) tool EDDY_CORRECT was used to correct for eddy-current and motion-related artifacts, including an appropriate correction of the diffusion gradient vector table. The FSL-tool DTIFIT was used to calculate the diffusion tensor as well as related measures such as fractional anisotropy. Additionally, individual T1 images were co-registered to their respective DWI data and non-linearly transformed to MNI space after skull stripping using FSL FLIRT and FNIRT. The combined inverse of the final non-linear transformation (DWI->T1->MNI) was created, allowing for a reverse-normalization of MNI space atlases or regions of interest into the individual subject space.

Probabilistic regions of interest of the corpus callosum subdivisions (BA4 upper limbs) depicting the connectivity between inter-hemispherical cortical primary motor areas were non-linearly transformed into subject space and assessed in terms of their weighted mean FA to generate an FA_UL mask (Domin and Lotze, in press).

Structural inter-hemispherical connectivity between the primary motor cortices was also calculated utilizing probabilistic tractography. We used FSL BEDPOSTX (Behrens et al., 2003) to infer the fiber orientation density function from the diffusion MRI with the subsequent application of FSL PROBTRACKX, which repetitively samples from the posterior distribution on principal diffusion directions and progresses along this same direction. A connectivity distribution is built by taking these samples, depicting a frequency map in which each voxel encodes the sum of streamlines running through that voxel. The M1 (left/right hemisphere) areas of the human motor area template (HMAT; Mayka et al., 2006) atlas were used as seed ROIs for tractography.

Finally, a weighted FA mean for each inter-hemispheric connection was calculated by masking each individual fractional anisotropy map with the corresponding tractogram.

### Volumetric Measures

T1-weighted images were preprocessed using SPM12 (v6225; Wellcome Department of Cognitive Neurology, University of London) and the CAT12 toolbox using default parameters (v1073^1^ Structural Brain Mapping Group, Jena University Hospital) and running on MATLAB (v8.4, The MathWorks, Natick, MA, USA). Images were first corrected for magnetic field inhomogeneities and segmented into cerebrospinal fluid, gray matter, and white matter. The segmentation process was refined using a hidden Markov Random Field model and accounting for partial volume effects, and finally the segmented images were spatially normalized using the DARTEL algorithm and individual gray matter volumes were calculated.

### Statistical Analysis

All statistical testing was performed using SPSS version 21. Data were normally distributed as determined with Kolmogorov-Smirnov tests, so we used parametric statistical procedures. The significance level was set at *p* < 0.05 after Bonferroni correction for multiple comparisons.

A one sample paired *t*-test was performed between the dominant and non-dominant hemisphere for the TMS measurements (ISP_onset, ISP_duration, ISP_depth, TCT).

One-sided Pearson correlations tested associations between age and TMS parameters (ISP_onset, ISP_duration, ISP_depth, TCT) to identify the most sensitive age-related parameter.

Pearson correlations were also performed between age and the DTI measurements (whole M1-M1, FA_UL) and brain volumes (individual gray matter volume, individual volume of corpus callosum).

The TMS and DTI-parameters most strongly correlated with age were then used to test associations between TMS and DTI measurements.

## Results

### Functional Neurophysiological Measurements

There were no significant differences in electrophysiological parameters between the hemispheres for RMT (*t*_(19)_ = −1.29, *p* = 0.2), AMT (*t*_(19)_ = 0.68, *p* = 0.5), ISP_duration (*t*_(19)_ = 1.32, *p* = 0.20), ISP_depth (*t*_(19)_ = 0.19, *p* = 0.85) and TCT (*t*_(19)_ = 1.04, *p* = 0.32). The ISP onset was delayed for non-dominant hemisphere stimulation (*t*_(19)_ = −2.3, *p* = 0.033; dominant hemisphere = 32.03 ± 5.82 ms, non-dominant hemisphere 35.15 ± 4.32 ms).

**Figure 1:**
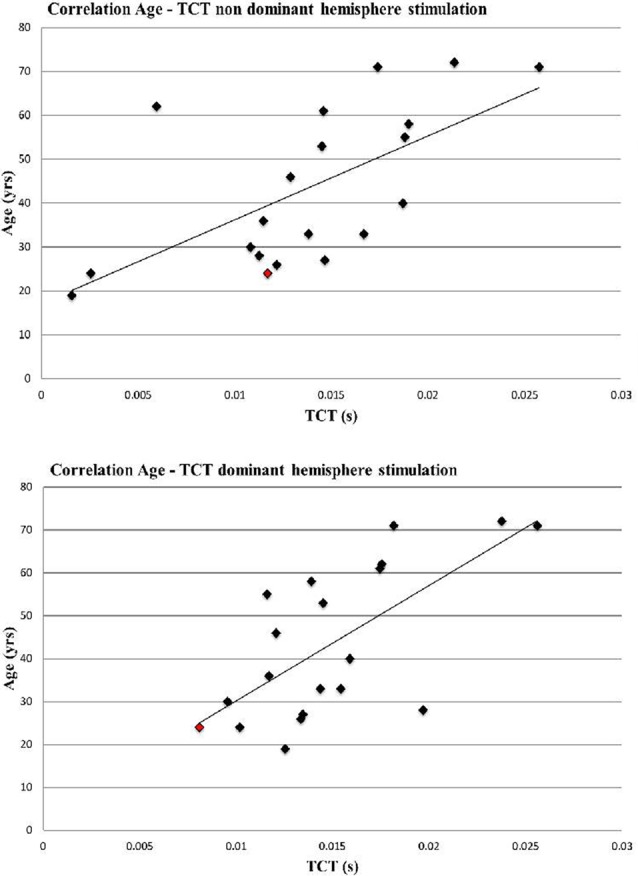
Correlation Age and transcallosal conduction time (TCT) of non-dominant and dominant hemisphere stimulation (non-dominant hemisphere stimulation: *r* = 0.63, *p* < 0.01; dominant hemisphere stimulation: *r* = 0.67 *p* < 0.01). Left handed participant is marked in red.

Only TCT correlated significantly with age (non-dominant hemisphere stimulation: *r* = 0.63, *p* < 0.01; dominant hemisphere stimulation: *r* = 0.67 *p* < 0.01; see Figure 1), and all other parameters (ISP_onset; ISP_duration; ISP_depth) were not significantly correlated (all *p* > 0.1).

### Structural Measurements

The mean gray matter volume was 714.5 ± 79 mm^3^, and there was a correlation between individual gray matter volume and age (gray matter: *r* = −0.48; *p* = 0.02).

**Figure 2:**
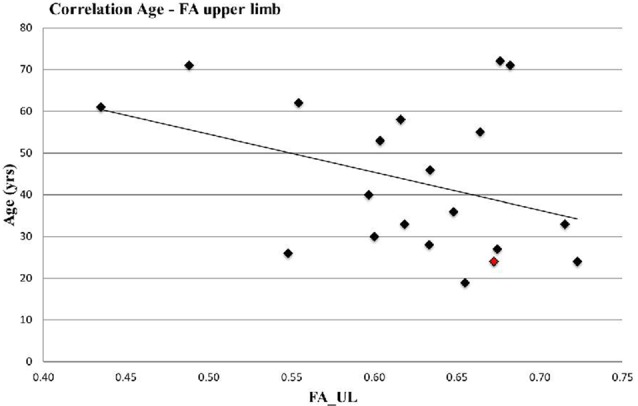
Correlation Age and Fractional Anisotrophy (FA)_UL (*r* = −0.37, *p* = 0.055). Left handed participant is marked in red.

While FA values for the whole transcallosal M1-M1 connection did not correlate with age (*r* = 0.17, *p* = 0.23), FA_UL showed a strong trend toward significance for a negative age association (*r* = −0.37, *p* = 0.055; Figure 2).

### Functional-Structural Relationship

**Figure 3:**
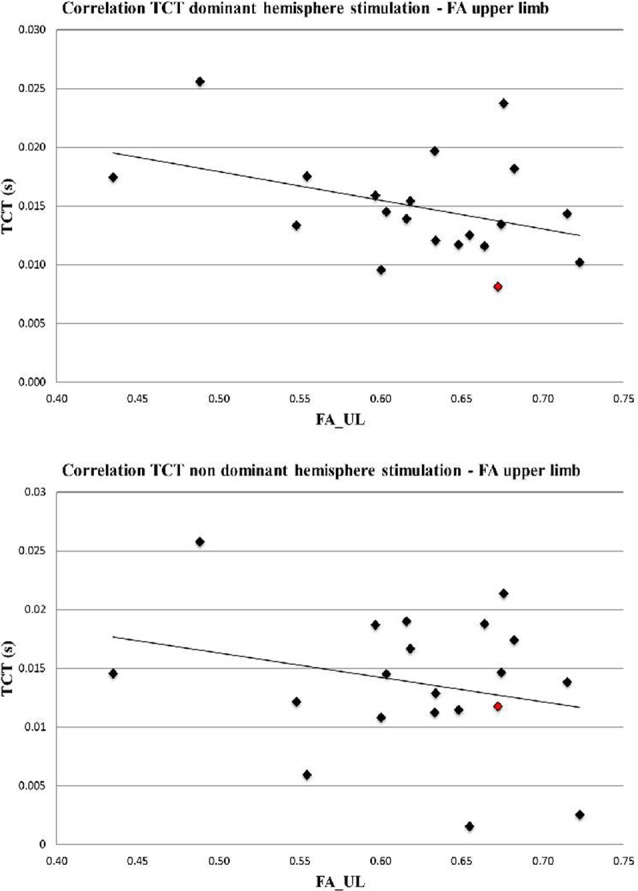
Correlation TCT and FA_UL (dominant hemisphere *r* = −0.39, *p* = 0.043, non-dominant hemisphere *r* = −0.25, *p* = 0.19). Left handed participant is marked in red.

TCT of the dominant hemisphere showed a significant negative correlation with FA (*r* = −0.39, *p* = 0.043), while no significant correlation emerged for the non-dominant hemisphere (*r* = −0.25, *p* = 0.19; Figure 3).

## Discussion

The aim of this study was to identify the parameters of functional and structural interhemispheric connectivity that were most strongly associated with age, and to determine if these parameters were altered consistently over the lifespan.

We found that age related decline in functional connectivity is best represented by the decline in the TCT, and structural connectivity by the upper limb part of the M1-M1 interhemispheric connectivity. Additionally, the functional- structural relationship was maintained over the lifespan only for the dominant hemisphere.

With regard to functional connectivity, several neurophysiological studies have compared interhemispheric inhibition between young and old participants, showing that older participants had delayed ISP onset, decreased ISP depth or area, and a prolonged TCT (Davidson and Tremblay, 2013; Petitjean and Ko, 2013; Coppi et al., 2014; Levin et al., 2014). Together these findings strongly support a decline in interhemispheric inhibition between M1 areas with age. This study confirms these results, and suggests that this decline is uniform across the lifespan.

Importantly, we assessed several parameters for interhemispheric inhibition (onset, depth and duration of ISP, TCT), all of which have been used in previous reports, albeit with inconsistent results (Chen et al., 2003; Fling and Seidler, 2012; Davidson and Tremblay, 2013; Coppi et al., 2014). Petitjean and Ko (2013) compared a group of young (mean 28 years) and old (mean 58 years) participants measuring various interhemispheric parameters and found that TCT showed the strongest difference between age groups for both hemispheres, as shown here. Davidson and Tremblay (2013) investigated TCT and ISP area in a young vs. old comparison and demonstrated that both parameters differed. Unlike ISP, TCT is adjusted for potential peripheral conduction impairment in the definition of MEP onset. If age-related delay was caused by peripheral conduction impairment (more likely for older than younger participants), we would expect the opposite effect, with later ISP rather than TCT onset in older compared to younger participants. Jung and Ziemann (2006) demonstrated that ISP but not TCT onset and duration varies between different intrinsic hand muscles, also arguing for the reliability of this parameter. In sum, based on the work by Petitjean, and the current findings of a linear decline of TCT with age, we recommend the use of TCT in future studies (Fling and Seidler, 2012; Davidson and Tremblay, 2013; Coppi et al., 2014).

With regard to structural interhemispheric connectivity, an extensive body of studies regarding gray and white matter changes across the lifespan has been published (Salat, 2011; Lockhart and DeCarli, 2014; Zhao et al., 2015; Bajaj et al., 2017), although less is known about white matter interhemispheric connectivity between motor areas. Here, inconsistent findings have been reported with regard to changes of integrity of white matter tracts with age, with some studies showing a positive (Inano et al., 2011), other studies a negative (Inano et al., 2011; Voineskos et al., 2012) correlation of DTI parameters for different callosal subregions. Varying correlations within different callosal subregions were also reported by Sullivan et al. (2010). Using fiber tracking in different tracts, they demonstrated that age was inversely associated with FA in all callosal sectors except the sensorimotor region and the splenium (Sullivan et al., 2010). Similarly, we also found no association for the transcallosal M1 tract (*r* = 0.17; *p* = 0.23). Importantly, we used a novel method for interhemispheric M1-M1 white matter tract parcellation to enhance the spatial resolution of the interhemispheric tract (Domin and Lotze, in press). Distinct subregions connecting both primary motor cortices like the tract for the upper limb motor pathways can be assessed in more detail using this method. We demonstrated that connectivity in the upper limb area of the M1-M1 white matter tract was associated with age, although this relationship did not reach significance (with *p* = 0.055), which in our opinion is due to an underpowered sample size. The association of small subregions but not of the whole M1 region again emphasizes the importance of using a more advanced method in future studies, and suggests a possible different association to age for different subregions of M1.

We next associated the functional and structural measurements of interhemispheric motor connectivity most strongly associated with age, i.e., the TCT and FA_UL.

TCT was associated with structural FA_UL, suggesting that conductivity of interhemispheric inhibition of the small hand muscles and the interhemispheric upper limb transcallosal fiber-tract represent different aspects of the same age-related decline. However, there was a difference between stimulation to the dominant and non-dominant hemisphere, and only stimulation to the dominant hemisphere was significant. Coppi et al. (2014) showed that ISP onset was prolonged in the non-dominant compared to dominant hemisphere and for old compared to young participants, which is in line with our results. This difference might be a reason for the significant association for the dominant but not for the non-dominant hemisphere in correlating the functional and structural interhemispheric connectivity, and suggests that the association with age might be more pronounced for the dominant hemisphere.

This study has several limitations. First of all, our sample size is rather small. Since data about structural connectivity usually comes from larger cohorts, our findings require additional confirmation. However, we here used a relatively new parcellation of interhemispheric tracts that will increase the effect size of this method helping to assess data even in smaller sample sizes. Additionally, previous neurophysiological studies comparing old and young participants had comparable sample sizes between 10 and 20 participants, and our sample size was sufficient to confirm an age related decline in functional interhemispheric motor connectivity (Sullivan et al., 2010).

Different methods with varying effect sizes complicate the interpretation of results. More elaborate procedures, such as those performed here by using a more precise parcellation of fiber tracts of the CC, or navigated TMS or TMS-based tractography are necessary to increase knowledge on structural-functional relationships.

Additionally, while functional measurements are bidirectional, structural interhemispheric connectivity can only be assessed unidirectionally and DTI does not allow for discrimination of path direction. Additionally, structural information does not include physiological information about facilitation or inhibition. However, our results are in line with others that could demonstrate this functional-structural association for the M1-M1 interhemispheric motor connectivity (Wahl et al., 2007; Fling et al., 2012) extending the knowledge for a homogenous age related decline.

In conclusion, we demonstrate an age-related decline in interhemispheric inhibition, as assessed with both functional and structural measurements, in the present cohort. By assessing several parameter of functional and structural connectivity, we were able to demonstrate that TCT and FA_UL, respectively, might be most suitable for future studies. Additionally, the functional-structural relationship was only statistically significant on the dominant hemisphere, suggesting a difference in age related decline in interhemispheric motor connectivity between the dominant and non-dominant hemisphere. More studies are needed to understand the mechanisms underlying these changes in interhemispheric connectivity, which might help to interpret the physiological and pathological alterations, and to develop targeted approaches to prevent or counteract these.

## Author Contributions

SS and MG conducted the TMS analyses and wrote the initial draft of the manuscript and organized the revisions. MD conducted the neuroimaging analyses. ML and AF each contributed to the writing of revisions of the manuscript.

## Conflict of Interest Statement

The authors declare that the research was conducted in the absence of any commercial or financial relationships that could be construed as a potential conflict of interest.

## Footnotes

^1^http://www.neuro.uni-jena.de/cat
